# The neck-region polymorphism of DC-SIGNR in peri-centenarian from Han Chinese Population

**DOI:** 10.1186/1471-2350-10-134

**Published:** 2009-12-14

**Authors:** Hui Li, Cheng-Ye Wang, Jia-Xin Wang, Nelson Leung-Sang Tang, Liang Xie, Yuan-Ying Gong, Zhao Yang, Liang-You Xu, Qing-Peng Kong, Ya-Ping Zhang

**Affiliations:** 1 State Key Laboratory of Genetic Resources and Evolution, Kunming Institute of Zoology, Chinese Academy of Sciences, Kunming 650223, PR China; 2 Laboratory for Conservation and Utilization of Bio-resource & Key Laboratory for Microbial Resources of the Ministry of Education, Yunnan University, Kunming 650091, PR China; 3 Graduate School of the Chinese Academy of Sciences, Beijing 100049, PR China; 4 KIZ/CUHK Joint Laboratory of Bioresources and Molecular Research in Common Diseases, Kunming 650223, PR China; 5 Li Ka Shing Institute of Health Sciences, The Chinese University of Hong Kong, Hong Kong; 6 Department of Chemical Pathology, Faculty of Medicine, The Chinese University of Hong Kong; 7 People's Hospital of Dujiangyan, Dujiangyan 611830, PR China; 8 Dujiangyan Longevity Research Centre, Dujiangyan 611830, PR China

## Abstract

**Background:**

DC-SIGNR (also called CD209L) has been extensively studied on its role in host genetic predisposition to viral infection. In particular, variable number tandem repeat (VNTR) of the neck-region of DC-SIGNR is highly polymorphic and the polymorphism has been investigated for genetic predisposition to various infectious diseases, though conflicting results had been reported. As infection is a major cause of human death and a mechanism of natural selection, we hypothesized that VNTR polymorphism of DC-SIGNR might have an effect on human life span.

**Methods:**

Here we collected 361 peri-centenarian individuals (age ≥94 for female and age ≥90 for male) and 342 geographically matched controls (age 22-53, mean 35.0 ± 12.0) from Han Chinese. The VNTR polymorphism of the neck region was determined by PCR and genotype was called by separating the PCR products in agarose gel.

**Results:**

A total of 11 genotypes and 5 alleles were found in our population. The genotype distribution, allele frequencies and homozygote proportion did not show a significant difference between peri-centenarian and control group. As gender differences in lifespan are ubiquitously observed throughout the animal kingdom, we then stratified the samples by gender. There was more 6/7 genotypes in female peri-centenarian group than that in female control group, at a marginal level of significance (5.56 vs. 1.28%, p = 0.041). The difference was not significant after correction by Bonferroni method. It suggests a possible differential effect of DC-SIGNR VNTR genotypes between sexes. Further studies are warranted to confirm our preliminary findings and investigate the mechanisms of the underlying functions.

**Conclusions:**

Our study indicated that there was absence of association between the neck region polymorphism of DC-SIGNR and longevity in Han Chinese population. But the question of whether the DC-SIGNR could affect longevity in a gender-specific pattern remains open.

## Backgroud

Dendritic cell-specific intracellular adhesion molecular-3-grabbing nonintegrin (DC-SIGN) and DC-SIGN related (DC-SIGNR) are C-type lectins involved in both innate and adaptive immunity. As DC-SIGN and DC-SIGNR originated from the duplication of the same ancestral gene, they are located side-by-side within a ~26-kb segment of chromosome 19p13.2-3 and they share similar functions as cell adhesion receptors and pathogen recognition receptors[1]. A large number of their binding targets are of clinical significance, including bacteria such as *Mycobacterium tuberculosis*, *Helicobacter pylori*, and certain *Klebsiela pneumonia *strains, as well as virus, such as HIV-1, HIV-2, Ebola virus, cytomegalovirus, hepatitis C virus(HCV), Dengue virus, and SARS-coronavirus [2-5]. One difference between DC-SIGN and DC-SIGNR is that DC-SIGN is expressed primarily on phagocytic cells, such as dendritic cells and macrophages, whereas DC-SIGNR expression is restricted to endothelial cells in liver, lymph nodes and placental capillaries[6,7].

DC-SIGNR is a type II transmembrane protein composed of three domains: an N-terminal cytoplasmic domain, a neck region made up of a highly conserved 23-amino-acid repeat, and a C-terminal extracellular C-type carbohydrate recognition domain (CRD) involved in pathogen binding[8]. The neck region connects the CRD from the transmembrane region and assembles the lectin into a tetrameric protein conformation on the cell surface. Thus the neck region is responsible for the homo-oligomerization that brings the CRDs into proximity for high-affinity ligand binding[9]. On the other hand, heterozygous expression of DC-SIGNR of different length in the neck region may lead to reduced ligand-binding affinity and loss of the efficient recognition of multivalent ligands[8,10]. Furthermore, the length variations of this neck region can critically influence the pathogen-binding properties of the CRD of these proteins, as some studies have found associations between length variation of the neck region and susceptibility to various infectious diseases, such as HIV, HCV, and SARS [11-17], though conflicting results were also found[18]. These infectious diseases affect an individual's life span, since they can be life-threatening and may lead to early death if lacking effective treatments. This is one of the mechanisms for natural selection in a population.

It has been suggested that polymorphisms in immune-related genes not only affect susceptibility to infectious diseases[19], but also influence the aging process through immunosenescence[20]. Because the highly polymorphic DC-SIGNR neck region plays such a vital role in host genetic predisposition to a number of infectious diseases, we hypothesized that it would also influence the aging process and might be a genetic factor for longevity. To confirm this hypothesis, we collected 361 unrelated peri-centenarian individuals (age ≥94 for female and age ≥90 for male) and 342 normal controls (age 22-53, mean 35.0 ± 12.0) in Han Chinese population, and genotyped their VNTR polymorphism of DC-SIGNR neck region. To the best of our knowledge, this is the first study on the association between VNTR polymorphism in DC-SIGNR gene and longevity.

## Methods

### Subjects

This elderly Han Chinese cohort composed of 485 unrelated peri-centenarian individuals (age ≥94 for female and age ≥90 for male; mean age 95.14 ± 2.40) were recruited from Dujiangyan in Sichuan province of China. As reported in previous publication[21], ages of the subjects were authenticated by the official certification of the Fifth Nation Census in China which was also supported by information of the number of offspring generations (≥3), and local village records. Only subjects with their age supported by both government identity record, local village record and fulfilled the required number of offspring generation were included.

An interview was carried out for each peri-centenarian subjects at their homes by physicians during a home visit session. A brief medical history and examination was carried out to exclude participants with clinically apparent diseases (such as chronic lung disease, chronic heart failure, severe vision disorders, severe hearing disorders, hypertension, fracture, and arthritis severly limiting activity of daily living). More than 500 peri-centenarian individuals (age ≥94 for female and age ≥90 for male) have been interviewed but only 485 individuals (246 males and 239 females) who fulfilled all inclusion criteria, were included into this peri-centenarian cohort.

As we have more samples to fit into four 96-well plates for genotyping (each plate holds 90 samples while the reminding wells were used for QC samples and negative control, i.e. 360 were genotyped in these four 96-well plates), we blindly pull out a block of 180 male and 180 female samples for genotyping. One additional male sample was used for reaction optimization and his genotype results was also included, which led to a total of 181 male genotyping results.

Meanwhile, 342 healthy younger individuals, which included 156 females and 186 males, were also recruited from the same area, to serve as a control group (aged 22-53, mean age 35.0 ± 12.0). The demographic characteristics of these samples have been described in our previous work[21]. All participants signed informed consent after they had been given a clear explanation of the potential risk of the study according to the Declaration of Helsinki. This research has been approved by the Ethics Committee on human experimentation of Kunming Institute of Zoology, Chinese Academy of Sciences and institutions in Dujiangyan.

### Genotyping and Statistical Methods

Peripheral blood was collected from the participants and genomic DNA was extracted using the standard phenol/chloroform method. The VNTR polymorphism of the neck region was genotyped using the assay described in Chan *et al. *[12]. The genotype was determined by separating the PCR products in 3% agarose gel with ethidium bromide. To validate the genotyping results, 10% of the samples were re-genotyped by either duplicated genotyping experiments or direct sequencing.

Hardy-Weinberg equilibrium (HWE) test for VNTR in the peri-centenarian and control groups was performed by GENEPOP software http://wbiomed.curtin.edu.au/genepop/index.html. Homozygote proportions, genotype distribution and allele frequencies were compared by two-tailed chi-square test or Fisher's exact test (SPSS 11.5, SPSS Inc) (df = number of genotypes being analysed minus 1). When *P *value < 0.05, it was considered to be statistically significant and further subjected to Bonferroni correction for multiple testing.

### Sample size determination

Allele and genotype distribution of encoding VNTR in DC-SIGNR gene in Chinese populations of different parts of China have been surveyed and the data have been published in our previous work[18]. Based on the frequencies of various alleles of the VNTR (Additional File 1: supplementary table S1), the sample size (number of chromosomes) required to show an allelic odd ratio of 1.5 and 2 were determined by the program STATCALC (CDC, USA). For example, the 5-repeat allele was found at an allelic frequency of 0.16 in Han population. A sample of 657 cases and 657 controls chromosomes is required to show an effect caused by an allelic odd ratios of 1.5 at power of 80% at a 2-sided α value of 0.05. Details of sample size required in various scenario of association allele and odd ratios are shown in Additional file 1: supplementary table S2. It was found that, for most of the prevalent VNTR alleles, our sample has sufficient power to detect an association with allelic odd ratios of 2, except for the allele of 8-repeats, which was found at a MAF of 0.002.

## Results and Discussion

We genotyped the VNTR polymorphism of DC-SIGNR neck region among 361 peri-centenarian individuals and 342 matched young individuals in Han Chinese. The homozygote proportion, genotype and allele frequencies of the VNTR were listed in Table 1. A length variation ranged from 5 repeats to 9 repeats in this VNTR locus were observed in our samples, and these different repeats (alleles) form 11 genotype. The most common genotype in our population is 7/7 (homozygote for the allele of 7-repeats), followed by 5/7 and 7/9. The allele 7 has the highest frequency of 66.6%, followed by allele 5 of 15.5%, allele 9 of 14.15% and allele 6 of 3.62%. The allele 8 was fairly rare in Han Chinese population with a frequency of only 0.13%. The genotype frequencies of peri-centenarian and control population did not deviate from the Hardy-Weinberg equilibrium (p > 0.05, by both chi-square and a Markov chain method in GENEPOP). Our results indicated that there was no significant difference in the homozygote proportion, genotype distribution and allele frequencies between the two groups. While, as we know, animals from a broad variety of taxa show sex differences in lifespan, for example, in mammals and insects, females commonly live longer than males[22,23], but males outlive females in most bird taxa[24]. To investigate whether there is a potential difference in our samples introduced by gender, we further divided the two groups into four: female peri-centenarian, male peri-centenarian, female control and male control. All groups also followed the Hardy-Weinberg equilibrium. The result indicated that there was more 6/7 genotype in female peri-centenarian group than that in control group (Table 2) with a marginal level of significance (5.56 vs. 1.28%, p = 0.041), but this difference was no longer significant after correction by Bonferroni method. It seems that the 6/7 genotype may show a false positive association due to multiple testing. However, we cannot exclude a functional role of 6/7 genotype and the hypothesis that the effect of DC-SIGNR on longevity maybe different between males and females. Considering the relative small sample size of this study, our hypothesis needs to be replicated with larger sample size.

**Table 1:** Genotype, allele distribution and homozygote proportion of DC-SIGNR neck region in peri-centenarian and control group.

	Peri-centenarian		Controls		
Genotype	(N = 361)		(N = 342)		*P*
5/5	9	2.49%	10	2.92%	0.082
5/6	3	0.83%	5	1.46%	0.495
5/7	70	19.39%	73	21.35%	0.574
5/9	15	4.16%	14	4.09%	1.000
6/6	2	0.55%	0	0.00%	0.500
6/7	15	4.16%	15	4.39%	1.000
6/9	3	0.83%	6	1.75%	0.329
7/7	165	45.71%	151	44.15%	0.705
7/8	0	0.00%	1	0.29%	0.486
7/9	70	19.39%	61	17.84%	0.629
9/9	9	2.49%	6	1.75%	0.605
homozygotes	185	51.25%	167	48.83%	0.546
heterozygotes	176	48.75%	175	51.17%	0.546
					
Alleles					
5	106	14.68%	112	16.37%	0.418
6	25	3.46%	26	3.80%	0.776
7	485	67.17%	452	66.08%	0.692
8	0	0.00%	1	0.15%	0.486
9	106	14.68%	93	13.60%	0.592

**Table 2:** Genotype, allele distribution and homozygote proportion of DC-SIGNR neck region in peri-centenarian and control group stratified by gender.

	Peri-centenarian Female		Controls Female			Peri-centenarian Male		Control Male		
Genotype	N = 180		N = 156		*p*	N = 181		N = 186		*p*
5/5	1	0.56%	6	3.85%	0.053	8	4.42%	4	2.15%	0.253
5/6	1	0.56%	3	1.92%	0.341	2	1.10%	2	1.08%	1.000
5/7	29	16.11%	32	20.51%	0.322	41	22.65%	41	22.04%	0.901
5/9	8	4.44%	3	1.92%	0.232	7	3.87%	11	5.91%	0.470
6/6	2	1.11%	0	0.00%	0.501	0	0.00%	0	0.00%	nc
6/7	10	5.56%	2	1.28%	0.041	5	2.76%	13	6.99%	0.089
6/9	2	1.11%	3	1.92%	0.666	1	0.55%	3	1.61%	0.623
7/7	83	46.11%	74	47.44%	0.827	82	45.30%	77	41.40%	0.463
7/8	0	0.0%	1	0.64%	0.464	0	0.00%	0	0.00%	nc
7/9	41	22.78%	29	18.59%	0.419	29	19.02%	32	17.20%	0.781
9/9	3	1.67%	3	1.92%	1.000	6	3.31%	3	1.61%	0.332
homozygotes	89	49.45%	83	53.21%	0.513	96	53.04%	84	45.16%	0.144
Heterozygotes	91	50.55%	73	46.79%	0.513	85	46.96%	102	54.84%	0.144
										
Alleles										
5	40	11.11%	50	16.03%	0.069	66	18.23%	62	16.67%	0.627
6	17	4.72%	8	2.56%	0.157	8	2.21%	18	4.84%	0.071
7	246	68.33%	212	67.95%	0.934	239	66.02%	240	64.52%	0.699
8	0	0.00%	1	0.32%	0.464	0	0.00%	0	0.00%	nc
9	57	15.83%	41	13.14%	0.381	49	13.54%	52	13.98%	0.915

Abbreviation: nc, not calculated.

Genetic factors are crucial in the ageing process and thus, also influence longevity. Mitchell et al. estimated a 25% heritability for the life-span in an Amish population in Lancaster County, Pennsylvania[25], which implied a relatively strong genetic impact on human life-span. However, many genetic factors and signaling pathways involved in ageing process are still unknown[26,27]. Here we hypothesized that genes which control immune response to infectious disease are likely related with longevity, but immune-related genes usually present a very high polymorphism and thus are prone to be confounded by population stratification in regular case-control studies[28]. For example, our former study suggested that a previously unrecognized population stratification, which was a geographic difference in genotype proportions and allele frequencies of the neck region of DC-SIGNR, existed in the Chinese of different geographic areas[18]. However, in the present study, our peri-centenarian and control groups came from the same community, and were ethnically matched, thus the spurious association caused by population stratification could be ruled out. Furthermore, all participants shared the same living environment and common diet habits, thus non-genetic factors were averted to the greatest extent.

A total of 11 genotypes and 5 alleles in neck region tandem repeat of DC-SIGNR were investigated in this population. The distributions of genotype and allele frequencies were similar to previous reports on Han Chinese populations[11,29]. Although there were reports suggesting a balanced selection could sharpen the alleles frequencies of this locus[30], the absence of association between the neck region polymorphism of DC-SIGNR and longevity in Han Chinese population may be caused by various reasons. First, most of the peri-centenarian individuals in this study had lower chance to get infectious diseases, as they were collected from Dujiangyan in Sichuan province, a relative isolated and remote region, i.e. the selection pressure on this locus was relaxed. For example, the sudden outbreak of SARS-CoV in 2003 was extensive in China but did not spread to this region [31,32]. Furthermore, the HIV-1 case was rare in the region we studied. In this case, many infectious diseases could not exert selective pressure on such population. This may be one explanation as to why we could not find an association between the neck region polymorphism of DC-SIGNR and longevity.

Another explanation for our result might be the relative small sample size in our study, especially when the samples were stratified by gender, which is also a handicap in many association studies. Therefore, replication with a larger sample size may improve our understanding on the consequence of this polymorphism and provide more explorative data about the potential influence of this gene on human longevity. After all, it was equally likely that DC-SIGNR gene did not have a role in healthy aging of human beings.

## Conclusions

We studied the association between VNTR polymorphism of DC-SIGNR neck region and longevity among peri-centenarian individuals in Han Chinese. Our results did not imply that the VNTR polymorphism was associated with longevity in our population. While, there was more 6/7 genotypes in female peri-centenarian group than that in female control group, with a marginal level of significance (5.56 vs. 1.28%, p = 0.041). The question of whether the DC-SIGNR could affect longevity in a gender-specific pattern remains unknown.

## Competing interests

The authors declare that they have no competing interests.

## Authors' contributions

HL, CW and YZ conceived the idea of the study and drafted the manuscript. JW conducted the genotyping of the VNTR and statistical analysis. NT assisted in revising the manuscript. LX, YG, ZY and LX collected the blood samples. YZ and QK supervised the study and obtained the funding. All authors read the paper and approved the final version.

## Pre-publication history

The pre-publication history for this paper can be accessed here:

http://www.biomedcentral.com/1471-2350/10/134/prepub

## Supplementary Material

Additional file 1 **Supplementary tables**. Table S1. Allele and Genotype distribution of encoding VNTR in DC-SIGNR gene in Chinese population samples collected from different parts of China. Table S2. Supplementary table of sample size required to detect an association caused by alleles at various allelic frequencies and allelic odd ratios. Click here for file
