# Supplementary material for: The neck-region polymorphism of DC-SIGNR in peri-centenarian from Han Chinese Population
Source: BMC Med Genet. 2009 Dec 14;10:134. doi: 10.1186/1471-2350-10-134 (PMC2797785; doi:10.1186/1471-2350-10-134)
Supplement: Additional file 1 — Supplementary tables. Table S1. Allele and Genotype distribution of encoding VNTR in DC-SIGNR gene in Chinese population samples collected from different parts of China. Table S2. Supplementary table of sample size required to detect an association caused by alleles at various allelic frequencies and allelic odd ratios. [file 1471-2350-10-134-S1.DOC]

**Supplementary tables.**

Table S1. Allele and Genotype distribution of encoding VNTR in DC-SIGNR gene in Chinese population samples collected from different parts of China

|  | **Southern China**(N=615) | | **Northern China**(N=530) | |
| --- | --- | --- | --- | --- |
| **Genotypes** |  |  |  |  |
| **4/7** | 0 | 0.0% | 0 | 0.0% |
| **5/5** | 17 | 2.8% | 14 | 2.6% |
| **5/6** | 4 | 0.7% | 2 | 0.4% |
| **5/7** | 116 | 18.9% | 114 | 21.5% |
| **5/9** | 29 | 4.7% | 24 | 4.5% |
| **6/6** | 3 | 0.5% | 1 | 0.2% |
| **6/7** | 40 | 6.5% | 24 | 4.5% |
| **6/9** | 5 | 0.8% | 7 | 1.3% |
| **7/7** | 277 | 45.0% | 234 | 44.2% |
| **7/8** | 1 | 0.2% | 1 | 0.2% |
| **7/9** | 110 | 17.9% | 89 | 16.8% |
| **8/9** | 0 | 0.0% | 0 | 0.0% |
| **9/9** | 13 | 2.1% | 20 | 3.8% |
| **homozygotes** | 310 | 50.4% | 269 | 50.8% |
| **heterozygotes** | 305 | 49.6% | 261 | 49.2% |
|  |  |  |  |  |
| **Alleles** |  |  |  |  |
| **5** | 183 | 14.9% | 168 | 15.8% |
| **6** | 55 | 4.5% | 35 | 3.3% |
| **7** | 821 | 66.7% | 696 | 65.7% |
| **8** | 1 | 0.1% | 1 | 0.1% |
| **9** | 170 | 13.9% | 160 | 15.1% |

Table S2. Supplementary table of sample size required to detect an association caused by alleles at various allelic frequencies and allelic odd ratios

| Allelic frequencies | Allelic odds ratio | Equal no. of cases and controls (chromosomes / no. of subjects) | Representing VNTR allele |
| --- | --- | --- | --- |
| 0.05 (5%) | 1.5 | 1774 / 887 | 6-repeat |
|  | 2 | 560 / 280 | 6-repeat |
| 0.16 (16%) | 1.5 | 658 / 329 | 5-repeat |
|  | 2 | 216 / 108 | 5-repeat |
| 0.65 (65%) | 1.5 | 476 / 238 | 7-repeat |
|  | 2 | 180 / 90 | 7-repeat |
